# Supplementary material for: Probing Mechanisms of Binding and Allostery in the SARS-CoV-2 Spike Omicron Variant Complexes with the Host Receptor: Revealing Functional Roles of the Binding Hotspots in Mediating Epistatic Effects and Communication with Allosteric Pockets
Source: Int J Mol Sci. 2022 Sep 29;23(19):11542. doi: 10.3390/ijms231911542 (PMC9569682; doi:10.3390/ijms231911542)
Supplement: Supplementary file 1 [file ijms-23-11542-s001.zip › ijms-1928626-supplementary.pdf]

Supplementary Information

Probing Mechanisms of Binding and Allostery in the SARS-CoV-2 Spike Omicron Variant Complexes with the Host Receptor: Revealing Functional Roles of the Binding Hotspots in Mediating Epistatic Effects and Communication with Allosteric Pockets

Gennady Verkhivker,[1,2]* Steve Agajanian[1], Ryan Kassab[1], Keerthi Krishnan[1]

[1] Keck Center for Science and Engineering, Graduate Program in Computational and Data Sciences, Schmid College of Science and Technology, Chapman University, Orange, CA 92866, United States of America
[2] Department of Biomedical and Pharmaceutical Sciences, Chapman University School of Pharmacy, Irvine, CA 92618, United States of America

* Correspondence: verkhivk@chapman.edu; Tel.: +1-714-516-4586 (G.V)

Received: date; Accepted: date; Published: date

Table S1. Statistical analysis of the intermolecular contact residues in Omicron RBD-hACE2 complexes.*

hACE2	BA.1 RBD	BA.1.1 RBD	BA.2 RBD	BA.3 RBD
S19	A475, G476,N477	A475, G476,N477	A475, G476,N477	A475, G476,N477
T20		N477	N477	A475,N477
Q24	A475, G476,N477 F486, N487, Y489	A475, G476,N477 F486, N487, Y489	A475, G476,N477 F486,N487, Y489	A475, G476,N477 F486,N487, Y489
T27	F456, Y473, A475,Y489	F456, Y473, A475,Y489	F456, Y473, A475,Y489	F456, Y473, A475,Y489
F28	N487,Y489	N487,Y489	N487,Y489	Y489
F30	L455, F456	N417, L455, F456	N417, L455, F456	L455, F456
K31	L455, F456, Y489,R493	L455, F456, Y489,F490, L492, R493	L455, F456, G485,Y489, R493	L455, F456, Y489,R493
H34	Y453, L455, R493, S494, Y495	N417, Y453, L455, R493	R403, N417, Y453, L455, R493	N417, Y453, L455, R493
E35	R493	R493	R493	R493
E37	H505	H505	H505	H505
D38	Y449, S496, R498, Y501	Y449, R493, S494, Y495, S496, R498, Y501	Y449, Y495, G496, R498, Y501	Y449, Y495, R498, Y501
Y41	R498, T500, Y501	R498, T500, Y501	R498, T500, Y501	R498, T500, Y501
Q42	S446, Y449, R498	Y449, R498	Y449, R498	Y449, R498
L45	R498,T500	V445, R498,T500	V445, R498,T500	V445, R498,T500
L79	F486	F486	G485,F486	F486
M82	F486	F486	F486	F486
Y83	F486, N487, Y489	F486, N487, Y489	F486, N487, Y489	F486, N487, Y489
Q325	V593		Q506	V503, Q506

G326				T500
N330	T500	T500	P499,T500	P499,T500
G352			Y501,G502	Y501,G502
K353	R403, Y495, S496, T500, Y501, G502, H505	Y495,S496 T500, Y501, G502,H505	R403, Y495, T500,Y501, G502, V503, H505	R403, Y495, T500,Y501, G502, V503, H505
G354	T500,Y501, G502,V503, H505	T500, Y501, G502, H505	T500,Y501, G502,V503, H505	T500,Y501, G502,V503, H505
D355	T500, Y501,G502	T500, Y501,G502	T500, Y501,G502	T500, Y501,G502
R357		T500	T500	

*Two residues are defined in contact if any of their heavy atom is within a distance of 5.0 Å

Table S2. The Occupancy of the Pairwise Interactions in the Omicron RBD-hACE2 Complexes

	Interaction	BA.1-ACE2	BA.1.1-ACE2	BA.2-ACE2	BA.3-ACE2
Salt bridges	R403-E37	65%	78%	73%	73%
	K440-E329	31%	56%	54%	54%
	R493-E35	77%	88%	92%	99%
	R493-D38	26%	95%	89%	89%
	R498-D38	59%	97%	95%	83%
Hydrophobic Interactions	F456-T27	95%	87%	96%	88%
	Y473-T27	92%	88%	89%	85%
	A475-T27	88%	87%	93%	83%
	F486-F28	78%	92%	97%	90%
	F486-L79	85%	93%	89%	82%
	F486-M82	85%	92%	96%	90%
	F486-Y83	90%	98%	95%	87%
	Y489-F28	97%	90%	94%	95%
	Y489-L79	90%	89%	95%	86%
	Y489-Y83	96%	78%	82%	88%
Hydrogen Bonds	Y453-H34	36%	42%	32%	92%
	Y449-D38	45%	54%	52%	58%
	A475-S19	30%	70%	65%	85%
	N477-S19	28%	72%	77%	97%
	N487-Y83	42%	76%	82%	92%
	T500-D355	62%	74%	77%	90%
	T500-Y41	62%	82%	80%	95%
	G502-K353	78%	82%	84%	78%
	Y501-K353	66%	92%	90%	84%
Specific Interactions	Y501-Y41	85	88%	92%	91%
	Y501-K353	82%	88%	96%	92%

Table S3. The electrostatic polar energies and desolvation energies for the RBD residues based on the equilibrium structure of the Omicron RBD BA.1-hACE2 complex.*

Residue Name	pKa	Elec.polar G(charged)	Elec. polar G(neutral)	Desolvation G(charged)	Desolvation G(neutral)
ARG0346	12.19	-0.1359	0.0049	0.0361	0.0050
ARG0355	12.97	-0.1693	0.0852	0.0967	0.0211
LYS0356	11.01	-0.1282	0.0403	0.0381	0.0060
ARG0357	12.26	-0.0860	0.0602	0.0467	0.0081
ASP0364	3.80	-0.2422	-0.0248	0.0870	0.0284
LYS0378	10.80	-0.0449	0.0139	0.0162	0.0076
LYS0386	10.87	-0.0564	-0.0173	0.0086	0.0044
ASP0389	3.92	0.0681	0.0532	0.0841	0.0318
ASP0398	2.74	0.1148	0.0403	0.2053	0.0562
ARG0403	13.09	0.2182	0.0144	0.1298	0.0228
ASP0405	2.85	-0.2475	0.0219	0.0930	0.0287
GLU0406	2.41	-0.5120	-0.1416	0.1728	0.0814
ARG0408	12.74	-0.1853	0.1030	0.0618	0.0084
ASP0420	3.16	-0.2342	-0.0468	0.1100	0.0307
LYS0424	11.22	-0.2218	-0.0781	0.0839	0.0091
ASP0427	3.49	-0.1535	0.0115	0.0506	0.0206
ASP0428	3.59	-0.3138	-0.0418	0.0885	0.0312
LYS0440	10.79	0.0707	0.0037	0.0147	0.0045
ASP0442	2.31	-0.1857	0.0955	0.1677	0.0415
LYS0444	10.48	0.0411	-0.0111	0.0447	0.0045
ARG0454	12.94	-0.8003	-0.1488	0.2183	0.0326
ARG0457	13.06	-0.2267	-0.0202	0.1139	0.0284
LYS0458	11.08	-0.1643	0.0370	0.0328	0.0053
LYS0462	10.93	-0.0337	0.0037	-0.0013	0.0051
GLU0465	3.06	-0.1407	0.0048	0.0779	0.0053
ARG0466	12.46	-0.5530	-0.2125	0.0913	0.0274
ASP0467	2.77	-0.3815	-0.0474	0.1338	0.0413
GLU0471	3.55	0.0021	0.0162	0.0232	0.0014
LYS0478	10.82	-0.0092	0.0056	0.0107	0.0046
ARG0493	12.97	-0.2343	0.0692	0.1424	0.0254
ARG0498	13.24	-0.3585	0.0178	0.1809	0.0226
HIS0505	5.98	0.1497	-0.0646	0.0853	0.0138
ARG0509	12.97	-0.3253	0.1119	0.1667	0.0291
GLU0516	3.79	0.0190	-0.0273	0.0752	0.0050
HIS0519	6.52	-0.0064	0.0024	0.0128	0.0039

* pKa value for each ionizable residue with associated energy terms (the unit here is kcal/mol). The electrostatic polar energy for individual residue in its protonated (+/-) state and neutral state, and the desolvation energy for individual residue in its protonated (+/-) state and neutral state.

Table S4. The electrostatic polar energies and desolvation energies for the RBD residues based on the equilibrium structure of the Omicron RBD BA.1.1-hACE2 complex.*

Residue Name	pKa	Elec. polar G(charged)	Elec. polar G(neutral)	Desolvation G(charged)	Desolvation G(neutral)
ASP0339	3.81	-0.0908	0.0711	0.0541	0.0211
GLU0340	3.46	-0.0170	0.0221	0.0496	0.0037
LYS0346	10.77	-0.0487	-0.0048	0.0129	0.0039
ARG0355	13.03	-0.2704	0.0674	0.1592	0.0396
LYS0356	11.07	-0.1082	0.0108	0.0445	0.0048
ARG0357	12.15	-0.0903	-0.0354	0.0661	0.0146
ASP0364	3.50	-0.5453	-0.2062	0.1596	0.3294
LYS0378	10.79	-0.0481	0.0023	0.0283	0.0063
LYS0386	10.76	0.2961	0.0784	0.0457	0.0072
ASP0389	3.97	0.2925	0.0105	0.0896	0.0277
ASP0398	2.69	0.2209	0.0373	0.2015	0.0707
ARG0403	13.00	0.2234	0.0133	0.1117	0.0170
ASP0405	2.79	-0.2302	0.0127	0.0779	0.0254
GLU0406	2.55	-0.2820	0.0030	0.1709	0.0287
ARG0408	12.56	-0.0134	0.0027	0.0388	0.0084
ASP0420	3.19	-0.2075	0.0623	0.1201	0.0455
LYS0424	11.52	-0.2984	0.0286	0.0807	0.0075
ASP0427	3.52	-0.1363	0.0068	0.0576	0.0222
ASP0428	3.79	-0.2309	-0.0258	0.0887	0.0318
LYS0440	10.79	0.0684	0.0046	0.0047	0.0050
ASP0442	2.50	-0.2114	0.1357	0.1722	0.0451
LYS0444	10.79	-0.1484	-0.0160	0.0476	0.0039
ARG0454	12.89	-0.5339	0.0827	0.1961	0.0331
ARG0457	13.07	-0.3141	-0.0174	0.1126	0.0206
LYS0458	11.04	-0.0493	0.0117	0.0201	0.0045
LYS0462	11.00	-0.0455	0.0027	0.0230	0.0053
GLU0465	2.95	-0.1673	-0.0385	0.0817	0.0064
ARG0466	12.49	-0.3143	0.0291	0.0609	0.0109
ASP0467	2.81	-0.3548	-0.0445	0.1265	0.0396
GLU0471	3.50	-0.0063	-0.0032	0.0246	0.0020
LYS0478	10.75	0.0994	-0.0091	0.0164	0.0053
ARG0493	13.62	-0.2106	-0.0522	0.1110	0.0240
ARG0498	12.55	-0.3407	-0.0812	0.1123	0.0127
HIS0505	6.02	0.1086	-0.0701	0.0749	0.0119
ARG0509	13.06	-0.4417	0.1141	0.1452	0.0320
GLU0516	3.50	-0.1058	-0.0044	0.0568	0.0027
HIS0519	6.71	0.1577	0.0439	0.0428	0.0138

Table S5. The electrostatic polar energies and desolvation energies for the RBD residues based on the equilibrium structure of the Omicron RBD BA.2-hACE2 complex.*

Residue Name	pKa	Elec.polar G(charged)	Elec. polar G(neutral)	Desolvation G(charged)	Desolvation G(neutral)
ASP0339	3.82	-0.0993	0.0421	0.0832	0.0277
GLU0340	3.05	-0.0686	0.0293	0.0811	0.0220
ARG0346	12.14	-0.0972	-0.0166	0.0460	0.0122
ARG0355	13.06	-0.1927	0.0874	0.1973	0.0739
LYS0356	11.20	0.0111	-0.0106	0.1754	0.1047
ARG0357	12.25	-0.3185	-0.1527	0.1040	0.0301
ASP0364	3.52	-0.5235	-0.0363	0.1045	0.0360
LYS0378	10.95	-0.2034	0.0732	0.0501	0.0055
LYS0386	10.87	0.0003	-0.0047	0.0097	0.0051
ASP0389	3.91	0.0406	0.0214	0.0672	0.0237
ASP0398	2.20	0.1758	0.0302	0.2426	0.1627
ARG0403	12.97	0.1549	0.0596	0.0951	0.0115
GLU0406	2.51	-0.3939	-0.1179	0.1513	0.0139
ASP0420	3.14	-0.3525	0.0029	0.1144	0.0308
LYS0424	11.82	-0.4666	0.0532	0.1300	0.0151
ASP0427	3.46	-0.1642	0.0409	0.0713	0.0250
ASP0428	3.61	-0.3245	-0.0656	0.0823	0.0297
LYS0440	10.83	-0.0327	-0.0003	0.0110	0.0049
ASP0442	2.21	-0.3310	0.1723	0.2134	0.0540
LYS0444	10.53	-0.0529	0.0196	0.0383	0.0046
ARG0454	13.09	-0.9663	-0.1073	0.2756	0.0419
ARG0457	13.22	-0.3610	0.0498	0.1616	0.0614
LYS0458	11.01	-0.0046	0.0223	0.0247	0.0053
LYS0462	10.99	-0.0913	-0.0080	0.0292	0.0077
GLU0465	2.79	-0.2246	-0.0936	0.1136	0.0122
ARG0466	12.50	-0.6164	-0.1977	0.0791	0.0173
ASP0467	2.92	-0.2084	-0.1494	0.1630	0.0588
GLU0471	3.46	-0.0586	0.0318	0.0229	0.0008
LYS0478	10.82	0.0146	0.0120	0.0075	0.0058
ARG0493	13.04	-0.3137	-0.1730	0.2313	0.0685
ARG0498	13.46	-0.6965	0.0474	0.2437	0.0374
HIS0505	5.78	0.0517	-0.0336	0.1003	0.0180
ARG0509	13.04	-0.4000	0.2088	0.2133	0.0454
GLU0516	3.26	-0.4601	-0.0451	0.0956	0.0168
HIS0519	6.50	0.0315	-0.0072	0.0120	0.0046

Table S6. The electrostatic polar energies and desolvation energies for the RBD residues based on the equilibrium structure of the Omicron RBD BA.3-hACE2 complex.*

Residue Name	pKa	Elec.polar G(charged)	Elec. polar G(neutral)	Desolvation G(charged)	Desolvation G(neutral)
ASP0339	3.78	-0.1619	0.0544	0.0680	0.0228
GLU0340	3.03	-0.0815	0.0242	0.0640	0.0342
ARG0346	12.15	-0.1075	-0.0152	0.0566	0.0178
ARG0355	13.04	-0.2072	0.0870	0.1497	0.0485
LYS0356	11.12	0.0344	0.0037	0.0519	0.0172
ARG0357	12.17	-0.1901	-0.2289	0.0486	0.0103
ASP0364	3.91	-0.0739	0.0332	0.1188	0.0345
LYS0378	10.85	-0.1407	0.0142	0.0316	0.0042
LYS0386	10.91	0.0002	-0.0080	0.0018	0.0043
ASP0389	3.83	0.0469	0.0261	0.1047	0.0339
ASP0398	2.50	0.1899	0.0363	0.2494	0.0886
ARG0403	13.13	0.0608	0.1371	0.0939	0.0155
GLU0406	2.22	-0.5186	-0.1393	0.1896	0.1051
ARG0408	12.21	-0.0873	-0.0165	0.0176	0.0039
ASP0420	3.32	-0.1717	0.0651	0.1037	0.0263
LYS0424	11.39	-0.2473	0.0059	0.1422	0.0105
ASP0427	3.50	-0.1628	0.0256	0.0613	0.0219
ASP0428	3.58	-0.3356	-0.0390	0.0924	0.0302
LYS0440	10.85	-0.1198	0.0033	0.0164	0.0037
ASP0442	2.10	-0.2708	0.0907	0.2122	0.0692
LYS0444	10.45	0.0291	-0.0179	0.0588	0.0063
ARG0454	12.99	-0.9878	-0.1701	0.3992	0.0710
ARG0457	13.20	-0.4717	-0.2062	0.1838	0.0345
LYS0458	11.02	0.0111	-0.0061	0.0520	0.0071
LYS0462	11.02	-0.0454	-0.0015	0.0271	0.0050
GLU0465	2.83	-0.2698	-0.0884	0.1026	0.0095
ARG0466	12.52	-0.6312	-0.1788	0.0898	0.0226
ASP0467	3.09	0.0036	-0.2796	0.1853	0.1827
GLU0471	3.42	-0.0223	0.0278	0.0305	0.0017
LYS0478	10.85	-0.0366	0.0047	0.0026	0.0057
ARG0493	13.00	-0.1220	0.0565	0.1390	0.0270
ARG0498	13.35	-0.6293	0.0144	0.2198	0.0332
HIS0505	5.76	0.0638	0.0190	0.0869	0.0140
ARG0509	12.99	-0.3698	0.1129	0.3041	0.0622
GLU0516	3.50	-0.2544	0.0114	0.1275	0.0238
HIS0519	6.52	-0.0329	0.0030	0.0136	0.0042
